# Vaccines for the Elderly and Vaccination Programs in Europe and the United States

**DOI:** 10.3390/vaccines12060566

**Published:** 2024-05-22

**Authors:** Cleo Anastassopoulou, Stefanos Ferous, Snežana Medić, Nikolaos Siafakas, Fotini Boufidou, Georgia Gioula, Athanasios Tsakris

**Affiliations:** 1 Department of Microbiology, Medical School, National and Kapodistrian University of Athens, 11527 Athens, Greece; sferous@med.uoa.gr (S.F.); atsakris@gmail.com (A.T.); 2 Department of Epidemiology, Faculty of Medicine, University of Novi Sad, 21000 Novi Sad, Serbia; snezana.medic@mf.uns.ac.rs; 3 Center for Disease Control and Prevention, Institute of Public Health of Vojvodina, 21000 Novi Sad, Serbia; 4 Clinical Microbiology Laboratory, Attikon General Hospital, Medical School, National and Kapodistrian University of Athens, 12462 Athens, Greece; nsiaf@med.uoa.gr; 5 Neurochemistry and Biological Markers Unit, 1st Department of Neurology, Eginition Hospital, Medical School, National and Kapodistrian University of Athens, 11528 Athens, Greece; fboufidou@med.uoa.gr; 6 Microbiology Department, School of Medicine, Aristotle University of Thessaloniki, 54124 Thessaloniki, Greece; ggioula@auth.gr

**Keywords:** immunization, older adults, herpes zoster, influenza, respiratory syncytial virus (RSV), COVID-19, pneumococcal disease, immunosenescence, vaccination programs

## Abstract

The share of the elderly population is growing worldwide as life expectancy increases. Immunosenescence and comorbidities increase infectious diseases’ morbidity and mortality in older adults. Here, we aimed to summarize the latest findings on vaccines for the elderly against herpes zoster, influenza, respiratory syncytial virus (RSV), COVID-19, and pneumococcal disease and to examine vaccine recommendation differences for this age group in Europe and the United States. PubMed was searched using the keywords “elders” and “vaccine” alongside the disease/pathogen in question and paraphrased or synonymous terms. Vaccine recommendations were also sought in the European and US Centers for Disease Control and Prevention databases. Improved vaccines, tailored for the elderly, mainly by using novel adjuvants or by increasing antigen concentration, are now available. Significant differences exist between immunization policies, especially between European countries, in terms of the recipient’s age, number of doses, vaccination schedule, and implementation (mandatory or recommended). Understanding the factors that influence the immune response to vaccination in the elderly may help to design vaccines that offer long-term protection for this vulnerable age group. A consensus-based strategy in Europe could help to fill the gaps in immunization policy in the elderly, particularly regarding vaccination against RSV and pneumococcus.

## 1. Introduction

### 1.1. The Aging of the Global Population

According to recently published data, global life expectancy at birth increased by approximately 23 years between 1950 and 2021, from 49.0 years (46.7–51.3) to 71.7 years (70.9–72.5) [1]. An interruption in these historical trends was noted during the coronavirus disease 2019 (COVID-19) pandemic period, when global life expectancy at birth declined by 1.6 years (1.0–2.2) between 2019 and 2021 [1]. Since 2017, a deceleration of population growth was recorded globally, except in a few lower-income countries; however, the global shift of population age structures toward older ages continued. In 2021, the worldwide population reached about 7.9 billion people and the proportion of the population aged 65 years, compared to those younger than 15 years, increased in 188 of 204 nations from 2000 to 2021 [1]. New challenges are presented to healthcare professionals by this demographic shift since the elderly represent a population especially vulnerable to disease and dysfunction [2].

### 1.2. The Aging of the Immune System

As the number of elderly people in the world increases rapidly, so does the morbidity and mortality due to infectious diseases in this age group. The reason for this is the gradual decline of the immune response associated with aging (termed “immunosenescence”) that is often exacerbated by the presence of comorbidities [3]. Starting in the sixth decade of life, the immune system ages and loses its ability to combat infections [4,5]. Consequently, the elderly present with more severe clinical disease forms and complications, and they have higher hospitalization and mortality rates from infectious diseases compared to younger people [4]. Febrile responses are frequently blunted in elders, and infections frequently present with nonspecific symptoms, making early detection of an infection a complicated process [6]. Additionally, an increased prevalence of malignancies and autoimmune diseases due to the augmented production of autoantibodies is associated with the aging of the immune system [7]. 

The functional capacity of the immune system to fight pathogens may become more limited over time due to increased systemic low-grade inflammation (inflamm-aging) and oxidation (oxi-inflammaging), as well as due to pathogen effects induced by persistent cytomegalovirus (CMV) infection and cellular senescence [3,7,8]. Among the many functions of the immune system that are impaired or reduced at an advanced age are phagocytosis, chemotaxis, and cytokine production, antibody generation, short-lived memory responses, naïve T- and B-cell pools, and cytotoxicity of natural killer (NK) cells [8]. In addition, T-cell exhaustion, i.e., lymphocyte proliferative senescence and various metabolic disturbances, including mitochondrial dysfunction, telomere shortening, and vitamin D deficiency, have been observed in older age [3,9]. 

Elevated levels of CRP, cytokines, and chemokines and abnormal white blood cell distribution in the elderly reflect a dysregulated inflammatory state associated with aging that contributes to the poor outcome of various infection diseases, including COVID-19 [10]. The degree of immune impairment varies between individuals of the same age and cannot currently be predicted by any specific markers, although recently, IL-6 and CRP have been established as the biomarkers most consistently associated with frailty in 20–30% of the elderly population [3,11].

### 1.3. The Need for Vaccine-Induced Immunity in the Elderly

**Figure 1:**
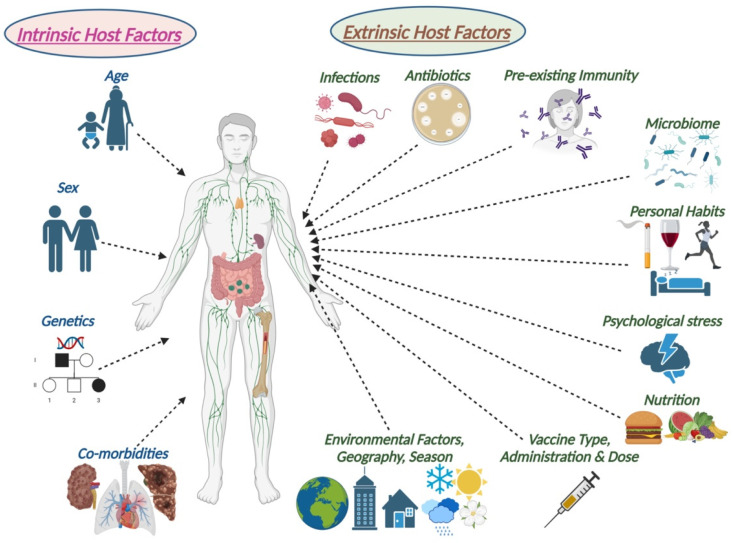
Intrinsic and extrinsic host factors that influence the immune response to vaccination. Adapted from [12] and created with BioRender.com.

Vaccination programs specifically targeted at immunizing those over the age of 60 can help prevent infection-associated morbidity and mortality [4]. A plethora of factors, both intrinsic and extrinsic to human hosts, influence the immune response to vaccination [12]. Apart from genetics and sex, age is an important intrinsic determinant of the immune response to vaccination (Figure 1). Both younger and older males and women of reproductive age are generally more prone to severe outcomes from respiratory viral infections such as influenza and COVID-19 [13]. Consistent sex differences also exist in response to vaccination or treatments. Women are more likely to develop greater immune responses but also more adverse reactions following administration of some viral vaccines due to more robust vaccine-induced cell-mediated and humoral immune responses. 

Lower vaccine-induced antibody levels coupled with a rapid decline in antibody levels over time contribute to a reduced immune response to vaccination in the elderly. Furthermore, a weaker cellular immune response to trivalent inactivated influenza vaccine (TIV), in particular, has been observed, which is partly related to a decline in the cytolytic capacity of CD8 T-cells responsible for clearing influenza virus from infected cells in old age [14]. Studies investigating intrinsic host factors influencing vaccine responses have found that comorbidities often associated with aging, such as diabetes, chronic renal or liver failure, and chronic cardiovascular or pulmonary diseases, may lead to reduction in vaccine immunogenicity due to lower vaccine-induced antibody immune response in the elderly [12]. 

In this respect, specific vaccine formulations catered to the needs of the weakened and frail immune system of the elders are necessary. The medical community and the pharmaceutical industry have responded to these challenges with the advancement of a number of elder-specific vaccines, such as the high-dose vaccine formulations against influenza that are specifically marketed for seniors [15]. Nonetheless, current knowledge on the real-world efficacy of available vaccines for elders is limited. Among its many other consequences, the COVID-19 pandemic had a tremendous impact on the routine vaccination against other pathogens [16], leaving elderly individuals vulnerable to other infectious agents. The pandemic might have also affected physician attitude and knowledge of vaccines available for the elderly [17].

To date, very few review articles have focused on vaccines that can help prevent morbidity and mortality among people aged 60 or 65 and older. A detailed review of vaccines against herpes zoster, influenza, and pneumococcal disease that were available for older adults in 2021 was provided by Cunningham et al. [4]. In this narrative review, we present and critically discuss current data on the employment of these three vaccines in the European Union (EU)/European Economic Area (EEA) and the United States (US) and also explore the scientific evidence for the best use of the novel vaccines against respiratory syncytial virus (RSV) and COVID-19 in the elderly. Background information that includes epidemiological, clinical, and immunological data is presented first for each pathogen and disease before presenting the evidence on available vaccines for older adults and comparing and contrasting current vaccine recommendations in the EU/EEA and the US. 

## 2. Methods

The PubMed database was searched for English-language original articles or reviews in peer-reviewed journals up to February 2024, using the medical subject heading (MeSH) terms “elders” and “vaccine” alongside the disease or pathogen in question, e.g., “herpes zoster” and “varicella zoster virus (VZV)”. Paraphrased or synonymous terms were also used to supplement the MeSH terms, including, for example, “immunization” or “vaccination” instead of “vaccine” and “older adults” or “seniors” instead of “elders” and “the elderly”. Publications in moderate- to high-impact Abridged Index Medicus (AIM) journals were selected. To explore the literature more effectively and to aggregate current data and provide a comprehensive review, we focused on more recent articles where possible, as well as on systematic reviews, meta-analyses, and randomized control trials. A historical perspective on the biology of aging and the development of vaccines for older adults is provided where needed, and results are generally presented in a chronological order. Vaccine recommendations for EU/EEA countries and the US were also sought in PubMed using the keywords “vaccination recommendations” and paraphrased terms, as previously described, for each studied pathogen and disease. Additionally, the latest recommendations for the vaccination of the elderly were sought on the European Centre for Disease Prevention and Control (ECDC) and the US Centers for Disease Control and Prevention (CDC) databases and their publications.

## 3. Results

### 3.1. Herpes Zoster Vaccines and the Elderly

#### 3.1.1. Epidemiology and Clinical Manifestations of Varicella and Herpes Zoster

Varicella zoster virus (VZV), a double-stranded DNA virus of the *Herpesviridae* family, is responsible for the development of varicella (chickenpox) upon primary infection and of herpes zoster (HZ, shingles) upon reactivation of a latent virus [18]. Most varicella cases are mild and occur by the age of 10 years in temperate climates [19]. Constitutional symptoms, such as fever, malaise, and loss of appetite, are frequently present; antiviral therapy is not recommended [20]. In adults, as well as in immunocompromised individuals, pregnant women, and newborns, varicella tends to be more severe [19]. Its characteristic pruritic rash, which consists of flat macules that evolve into fluid-filled vesicles, can even affect epithelial surfaces in the eyes and the genital tract [20]. The lesions subsequently become pustular, then dry out and form a dry crust [20]. After the rash has crusted over, patients usually cease to be contagious [19]. Following initial infection, VZV lays dormant in the trigeminal and dorsal root ganglia [21]. Latency in the nervous tissue following initial infection is primarily controlled by VZV-specific cell-mediated immunity [4].

Reactivation of the virus due to immune-compromise or any acute condition typically in older adults can result in the development of HZ, which usually affects a single dermatome [22,23]. HZ is commonly heralded by pain in the affected dermatome with associated pruritus. Subsequently, the characteristic herpetic rash appears [24]. Induced neuronal destruction and inflammation underlies HZ, which causes acute and chronic pain or post-herpetic neuralgia (PHN), significantly interfering with the quality of life of the elderly [25]. Although antiviral therapy with acyclovir does not seem to reduce the incidence of PHN [26], it can shorten the duration and severity of the episode that typically resolves within a few weeks. VZV reactivation and HZ occur more frequently in older adults, with the risk approximating 50% in individuals over 80 years of age [27]. 

The lifetime risk of HZ is estimated to be 10–30% but increases to 50% among those who live to ≥85 years [4,28]. The incidence of HZ depends on age and is ≈3 times higher in patients > 65 years (3.9–11.8 per 1000 persons per year) than in younger adults (1.2 to 3.4 per 1000 persons per year) [24], while the mortality rate related to HZ ranges from 0.0022 to 82.21 per 100,000 population [29]. PHN is a common long-term complication of HZ, defined as pain persisting for >90 days after the onset of the rash. The association between aging and PHN is undisputed. The percentage of HZ patients who develop PHN increases with age, as about 60% of patients aged 60 years and 75% of patients aged 70 years develop PHN following an acute HZ episode [30]. Other complications of HZ include ocular infections, encephalitis, myelitis, nerve palsy, and stroke, but the data about the frequency of these complications are scarce [31,32]. The incidence of HZ and the burden of disease due to complications, stress, and disability continue to increase, primarily in the elderly, as the population ages [33].

Live attenuated vaccines based on the *Oka* VZV strain that was developed by Takahashi in 1974 are available for the prevention of varicella [18]. In the United States, the vaccine against varicella has been used for the universal vaccination of children since 1995, a policy that led to a significant drop in the incidence of varicella [34]. However, since the varicella vaccine was not introduced until 1995, it is estimated that >95% of adults over 50 years old have been exposed to VZV and are at risk for VZV reactivation and HZ [35]. In the EU/EEA, varicella vaccination is not universally recommended; targeted varicella immunization for high-risk groups is implemented by most countries [36,37]. According to the recommendation of the World Health Organization (WHO), routine childhood immunization against varicella should be considered in countries where the disease has an important public health impact, if high vaccine coverage (≥80%) can be sustained [19]. Suboptimal varicella vaccine coverage levels <80% may lead to increased risks of severe disease and mortality in adults [19]. The availability of a tetravalent vaccine against measles, mumps, rubella, and varicella (MMRV) has led to a modification of varicella vaccination policies, mainly in developed countries.

#### 3.1.2. Immunology of VZV Latency

The seminal monograph of Hope-Simpson was one of the first works that associated HZ with increased age; it suggested that an age-associated decline in VZV-specific immunity was the cause behind VZV reactivation and HZ [38]. Clinical observations indicate that any decline in the function of cellular immunity is associated with more severe VZV infections and VZV reactivations, whereas isolated B-cell and antibody production deficits are not associated with increased VZV morbidity [39]. In addition, VZV-cellular responses decline with age, whereas antibody titers against VZV do not [40,41]. Therefore, the current scientific consensus is that VZV-cellular responses are responsible for establishing and maintaining latency, and the subsequent decline in cellular immunity as a normal part of aging is responsible for the rise in HZ in older adults [42]. In other words, VZV-specific cellular immunity, which consists of CD4+, CD8+ effector and memory cells, when functioning optimally, is adequate to maintain VZV-DNA latency [43].

Following any insult of cellular immunity, the latent genome of the virus is activated, resulting in the clinical manifestations of HZ. However, clinically silent reactivations of the virus, as defined by asymptomatic detection of VZV-DNA in the bloodstream, result in a boost of VZV-humoral and -cellular immune responses, a phenomenon termed “endogenous boosting” (compared to the “exogenous boost” observed when a VZV-immune individual is exposed to the wild-type virus or vaccine-type virus) [44]. Interestingly, it has been suggested that the universal uptake of varicella vaccination in children can result in the increase in zoster manifestations in young adults due to a decline in exogenous boosting [45,46]. The results of the recent study by Leung et al. do not support previous modeling predictions that the varicella vaccination program would increase the incidence of HZ among adult cohorts who experienced varicella. In contrast, a continued decline in age-specific HZ incidence is likely as varicella-vaccinated cohorts age [47]. More data from surveillance studies are needed to accurately determine the effect of universal varicella vaccination on the incidence of HZ in adults.

#### 3.1.3. Vaccines against VZV reactivation and Herpes Zoster

Considering the important role that cellular immunity plays in maintaining viral latency, a VZV vaccine must boost VZV-specific cellular immune responses for a substantial duration in order to effectively prevent VZV reactivation and HZ. Currently, two vaccine formulations against HZ are available: the recombinant zoster vaccine (RZV) and the zoster live vaccine (ZLV) [48]. ZLV is only approved for immunocompetent adults, and its use is contraindicated in immunocompromised individuals. It is no longer available in the USA; however, it is still used in other parts of the world, including Europe [49]. A single dose is provided, with no booster shots [50]. 

RZV consists of the viral glycoprotein E combined with the adjuvant AS01B [51]. In the United States, this vaccine combination has been approved for use in all immunocompetent adults over 50 years of age and in immunocompromised individuals over the age of 18 (Table 1). The vaccination schedule consists of two doses, with the second dose being given two to six months following the first one in all immunocompetent adults and one to two months after the first dose in immunocompromised individuals [49,50]. The immunization policy is much more complex in the 12 EU/EEA countries that recommend HZ vaccination for older adults (Table 1). Most countries with an HZ immunization policy in effect recommend vaccination for individuals older than 64 (Greece) or 65 years of age (Estonia, Luxembourg, and Spain), but in Italy and France, the recommendation is for those aged 65–74 years and 65–75 years, respectively. Cyprus recommends vaccination for individuals older than 60 years, and in Germany, the recommendation is only for people between the ages 60 and 64. Liechtenstein recommends targeted vaccination for specific groups older than 65 years. Surprisingly perhaps, the recommended vaccination for HZ for people older than 50 years in Austria and the Czech Republic or for people older than 60 years in Belgium is not funded by the respective national health systems.

#### 3.1.4. Efficacy of ZLV in the Elderly

The first major clinical trial that studied the efficacy of ZLV in individuals older than 60 years of age showed an overall efficacy rate of 51% in preventing HZ and an efficacy of 61.1% in reducing the severity of an HZ episode; a comparable efficacy of 66.5% in preventing the development of post-herpetic neuralgia was also found during this trial [52]. However, the study concluded that, when stratified for age, vaccine efficacy appeared to be lower in older individuals, with efficacy rates of 41% and 18% in individuals aged 70–79 and over 80 years of age, respectively [53]. Immunological studies of a subset of patients from the trial indicated that the magnitude of the immunological response following vaccination was less pronounced in older individuals, possibly due to higher Treg and Tcheck levels [54,55]. It is important to note that retrospective studies at the time did not corroborate the age-associated reduced efficacy shown in the original study [56]. A recent prospective study by Klein et al. showed a reduced overall efficacy when vaccine recipients were stratified by age, yet this effect was not as pronounced as in the initial study (47.6% effectiveness in preventing HZ in 50–59-year-olds vs. 41.4% in those over 80). The efficacy of the vaccine in preventing post-herpetic neuralgia was not affected by age [57].

The initial ZLV study indicated a lasting efficacy of the vaccine for 5 years [58], with initial phase I and II studies having also suggested a 5-year lasting efficacy before a statistically significant wane was noted [59,60]. Subsequent studies demonstrated a decline in efficacy after 5 years, with an estimated efficacy of 39.6% 8 years post-vaccination [61]. The same study by Klein demonstrated a reduction in vaccine efficacy over time for the development of both HZ and post-herpetic neuralgia, which could extend to 8 years post-vaccination, with only a minute amount of protection being present 10 years post-vaccination [57]. Booster shots received 10 years post-vaccination can increase anti-VZV cellular responses to levels higher than those achieved by the first dose [62]; thus, boosters could theoretically be offered to individuals over 70 years of age in order to increase vaccine coverage. Nevertheless, CDC guidelines recommend the administration of RZV in individuals who had received a previous injection of ZLV. RZV should be administered approximately 5 years after ZLV vaccination, but the interval could be shortened if the individual is over 70 years of age [49].

#### 3.1.5. Improving VZV Vaccination—The Use of RZV

The reduced effectiveness of the ZLV in elders, combined with the waning immune response, with virtually no protection present 8–10 years following vaccination, urged the medical industry to find different vaccine delivery platforms to augment VZV-cellular immunity for a substantial amount of time. Another important limitation of ZLV that had to be overcome is the contraindication of this vaccine for individuals with documented severe immunodeficiencies. 

Phase I and II studies indicated that a vaccine containing AS01B adjuvant in addition to the gE glycoprotein of the virus induced robust humoral and cellular immune responses that were not affected by the age of the vaccine recipient [63]. A subsequent booster shot further increased cellular and humoral responses by roughly 30%. This two-dose schedule was further studied in two clinical trials: one in a cohort of adults over 50 years of age (ZOE-50) and another in a cohort of adults over 70 years of age (ZOE-70). ZOE-50 found an efficacy of 97.2% in preventing HZ in the vaccination group. This efficacy rate was not diminished when stratified for age in contrast with the ZLV vaccine, maintaining an efficacy of over 96% across all age groups. Vaccine efficacy did not appear to wane during the course of the approximately 3 years of follow-up [64]. The ZOE-70 study was tailored to determine the efficacy of the vaccine in preventing HZ and the development of post-herpetic neuralgia in individuals over 70 years of age. ZOE-70 was conducted simultaneously with ZOE-50, and data from individuals over 70 that were randomized to participate in the ZOE-50 were also analyzed in ZOE-70. The ZOE-70 trial results supported the robust protection conveyed by the vaccine, in addition to the absence of an age-associated effect. There was no effect on the incidence of post-herpetic neuralgia following breakthrough HZ in vaccinated individuals compared to the placebo group [65]. A follow-up study further indicated the persistence of immunity for at least 7 years [66].

Few cohort studies have re-examined the effect of age on the effectiveness of the RZV. Sun et al. reported an overall vaccine efficacy of 83.5% in preventing HZ with 100%, 67.7%, 83.3%, and 86.4% efficacy in those aged 50–59, 60–69, 70–79, and over 80, respectively [67]. Meanwhile, Izurieta et al. reported an overall vaccine efficacy of 70% and an efficacy of 68.5% in individuals over 80 [68]. The substantially lower efficacy rates reported by Izurieta et al. compared to ZOE-50 and ZOE-70 could be attributed to the inclusion of immunocompromised patients in addition to the absence of individuals aged 50–64 [68]. The only study that demonstrated a reduction in vaccine efficacy in those older than 80 was a study by Sun et al., which showed an overall vaccine efficacy of 85.5% and an efficacy of approximately 80% in those over 80 [69].

According to the CDC, concomitant administration of RZV, at different anatomic sites, with other adult vaccines, including COVID-19 vaccines, is possible [49]. Studies on the coadministration of RZV with COVID-19 vaccines and adjuvanted influenza vaccine are underway. 

### 3.2. Influenza Vaccines and the Elderly

#### 3.2.1. Epidemiology and Clinical Manifestations of Influenza

Influenza is a respiratory illness, associated with a significant public health burden globally, due to seasonal epidemics caused by influenza A (A/H1N1pdm09 and A/H3N2) and B viruses (B/Victoria and B/Yamagata lineages) [4,70]. Influenza viruses are enveloped, single-stranded RNA viruses of the *Orthomyxoviridae* family, with a segmented genome [71]. The genomes of influenza A and B viruses are subject to frequent spontaneous mutations, especially on the viral surface proteins (hemagglutinin (HA) and neuraminidase (NA)), resulting in antigenic drift [72]; this process necessitates the deployment of yearly updated vaccines for the protection of the population based on globally circulating strains [73]. However, every 10–50 years, extensive genetic changes in influenza A viruses of zoonotic origin can lead to antigenic shift and the emergence of new viral strains of pandemic potential due to the lack of pre-existing immunity in humans [71]. Wild migratory birds are natural reservoirs of influenza A viruses that also infect domestic animals, pigs, poultry, horses, and many other species, including marine mammals, in addition to humans [71]. The wide circulation of influenza A viruses in nature in conjunction with the segmented nature of their genome allows for the interchange of pieces of genetic information between viral strains by reassortment after co-infection of the same host [71].

The burden of influenza varies from season to season, depending on the immunity of the population and the characteristics of the circulating strain of influenza virus [70]. In most immunocompetent individuals, influenza infection causes mild, self-limiting symptoms that include sore throat, rhinorrhea, cough, fever, and myalgias [74]. Complications include pneumonia, bacterial superinfection, and myocarditis. Severe influenza infections with subsequent complications and increased mortality are primarily observed in infants and the elderly, in addition to in immunocompromised individuals and pregnant women [74]. Several antivirals that target different stages of the life cycle of the viruses are available for the treatment of influenza, with Oseltamivir, a neuraminidase inhibitor that inhibits the release of virion progeny from infected cells, being the primary therapeutic agent [75].

The risk of influenza-related complications and hospitalizations is higher among older adults with underlying comorbidities [76]. Influenza is still a serious threat to the health of the elderly. Patients ≥65 years of age are at increased risk of severe disease and influenza-related complications due to immunosenescence and associated comorbidities [70,77]. It is estimated that more than 32 million adults become ill each year, resulting in 5.7 million influenza-related hospitalizations worldwide, with the highest hospitalization rates in people over 65 years of age [78]. Influenza causes 290,000–650,000 respiratory deaths annually [79], with estimated mortality rates of 50–100 per 100,000 in the age of 75 [80]. It has been estimated that 70–85% of influenza-related deaths and 50–70% of influenza-related hospitalizations during the 2010–2011 and 2019–2020 seasons were among those aged ≥65 years [81]. A wide range of respiratory complications associated with influenza, including primary viral influenza pneumonia and co-infection of influenza virus with bacteria that cause pneumonia (most commonly *Streptococcus pneumoniae* or *Staphylococcus aureus*) can lead to acute respiratory distress syndrome (ARDS), multiorgan failure, septic shock, and poor outcome [70].

#### 3.2.2. Immunology of Influenza and the Elderly

Four types of influenza viruses are known, A, B, C, and D, but only the first three have been found to infect humans, with influenza C viruses typically causing mild infections [71]. Therefore, influenza A and B are the major types associated with human illness and epidemics. Influenza A is characterized by the type of NA and HA present in the viral particle, with H1–H3 and N1 and N2, being the most common. Yearly vaccines are trivalent or quadrivalent primarily inactivated, split virion, or subunit vaccines that confer type-specific protection for the viral strains they were designed to target. For example, for the 2024–2025 vaccine formulations, the WHO selected H1N1 type A influenza, H3N2 type A influenza, and two B-type viruses from the Yamagata and Victoria lineages [82]. The primary immunogenic stimulus is provided by the HA molecules; however, NA stimulation might also act in an auxiliary fashion [83,84].

Strain-specific neutralizing antibody titers, as measured by HA-neutralization test assays, remain the primary method to infer protection [85]. A titer of at least 1:40 is considered protective and represents the titer at which approximately 50% of individuals are protected, provided that there is a good match between the circulating and vaccine strains [86,87]. Nevertheless, over the years, there have been reports of titers of the “protective level” that were not associated with protection [88]. 

Elderly individuals exhibit decreased phagocytic capabilities and reduced CD4^+^ activation that is associated with diminished CD8^+^ activity, in addition to a decline in B-cell populations [89]. These immune changes may explain, in part, the increased morbidity and mortality of influenza infections in elderly patients. Similarly, experimental studies have demonstrated that elderly individuals display an indolent and variable immune response following influenza vaccination [90]. The reduced pool and activity of B-cells result in reduced antibody responses following vaccination [91]. Consequently, elderly individuals exhibit a reduced responsiveness to influenza vaccination. Therefore, vaccine efficacy and possibly the duration of protection in elders is reduced, further increasing the overall risk for severe disease in this specific group [92,93]. In order to increase vaccine efficacy, augmented vaccine formulations were developed, including high-dose vaccines, adjuvanted vaccines, and recombinant hemagglutinin vaccines.

#### 3.2.3. High-Dose vs. Standard-Dose Influenza Vaccines 

Current high-dose vaccine formulations contain 60 μg of HA per strain, in contrast to the 15 μg contained in the standard vaccine [94]. Trivalent high-dose vaccinations became available in 2009; however, they were replaced by quadrivalent vaccines in 2019 [95]. Initial preclinical trials in healthy adults aged over 65 demonstrated a dose-dependent response following vaccination. Those who received the 60 μg dose systematically exhibit a higher mean and protective antibody titer [96,97,98]. Keitel et al. also showed that responses of higher magnitude were evident in those with lower pre-vaccination antibody titers, and that local injection site reactions were more common in the high-dose group [97].

Randomized studies on the efficacy of high-dose vaccines corroborated the pre-clinical findings and demonstrated that individuals who received the high-dose combinations had less severe infections and suffered from fewer hospitalizations and overall mortality [99,100,101]. Certain studies, however, have shown no statistically significant difference between high-dose and standard-dose vaccines [102]. In addition, although mild side effects with no lasting sequelae are more common in recipients of the high-dose combinations, the rate of serious adverse events that may result in disability, hospitalizations, or death in the high-dose group versus the standard-dose group are unlikely to be statistically significant [103]. 

#### 3.2.4. Adjuvanted vs. Standard-Dose Influenza Vaccines

Adjuvanted, inactivated subunit vaccines containing 15 μg of HA per strain in addition to the squalene-containing adjuvant MF59 have been approved since 1997 in the US and many other countries around the globe [104]. MF59 is presumed to augment immune function by prolonging antigen exposure, thus increasing antigenic stimuli, and by promoting recruitment of macrophages and dendritic cells at the intramuscular administration site, thereby stimulating pro-inflammatory cytokine production and subsequently boosting T- and B-cell activation [105]. An early study of an MF59-adjuvanted vaccine concluded that local, injection-associated reactions were more common in the adjuvanted group than in the standard vaccine group; however, most reactions were self-limited, and no severe adverse reactions were noted [106]. Although the study was not geared to assess immunogenicity, the adjuvanted group demonstrated a more robust immune response with higher antibody titers compared to standard vaccines [106]. Interestingly, the adjuvanted vaccines produce a broad antibody response capable of inducing protection even against heterologous strains [106], possibly by promoting the production of antibodies against the HA1 globular head instead of the HA2 domain [107]. The increased efficacy of MF-59-containing vaccines compared to standard-dosing vaccines have been reported by numerous studies, systematic reviews, and meta-analyses [108,109,110,111,112,113]. 

#### 3.2.5. Recombinant Hemagglutinin Vaccine

A tetravalent recombinant hemagglutinin vaccine, containing 45 μg of pure HA per strain, has also been approved for use in older individuals. The vaccine is produced by utilizing a baculovirus expression system, thus avoiding the use of egg-grown viral particles that have been associated with vaccine and circulating strain mismatch and reduced effectiveness [114]. 

A 2009 study by Keitel et al. compared the efficacy of a trivalent recombinant HA vaccine with a standard-dose trivalent in adults over 65 years of age and demonstrated that seroconversion rates were more pronounced in recombinant vaccine recipients, particularly those aged over 75 [115]. This effect was found for influenza type A but not for type B, possibly due to differences in the HA antigen contained in the two formulations. The side effect rate and severity were similar between the two groups [115].

However, a 2017 randomized control trial, which compared the efficacy of a recombinant tetravalent HA vaccine versus a standard-dose tetravalent vaccine during the 2014–2015 epidemic, which was characterized by vaccine and circulating strain mismatch, identified an overall recombinant vaccine efficacy in preventing influenza-like illness of 30% compared to the standard-dose formulation [116]. However, stratification for age revealed a relative vaccine efficacy of 42% in those aged 50–64 and only 17% in those over 65 when RT-PCR was used to determine breakthrough influenza infection [116]. Interestingly, the recombinant and the standard-dose vaccine were equally efficient in preventing clinical illness from the circulating influenza B strain. Adverse event rate and severity were similar between the two groups [116]. 

A 2023 retrospective study supported the findings by Dunkle et al., showing a higher relative recombinant vaccine efficacy in those under 65 years of age [117]. A different retrospective study by Zimmerman et al. reported no overall differences in efficacy between standard and recombinant vaccine formulations in preventing outpatient influenza illness [118]. Although the study did not include individuals over the age of 65, the recombinant vaccine was more effective in preventing mild illness in those aged 18–49 than in those aged 50–64 [118].

#### 3.2.6. Which Augmented Vaccine for the Elderly?

Most available randomized trials, in addition to most retrospective studies, compare the augmented vaccine formulations with the standard dose. Augmented vaccines are preferable to standard vaccines in those over the age of 65 due to their augmented immunological profile and efficacy [119]. A recent meta-analysis comparing the efficacy of high-dose influenza vaccines versus MF-59 vaccines, which analyzed data from 10 clinical studies, failed to demonstrate the superiority of one formulation over the other [120]. A 2021 retrospective analysis by Izurieta et al. concluded that all augmented vaccines were more effective than standard-dose vaccine in preventing hospital encounters due to influenza in those over 65, with the recombinant formulations being less effective than adjuvanted and high-dose formulations [121]. Clinical trials comparing augmented vaccines with each other have not been conducted as of yet.

The CDC’s Advisory Committee on Immunization Practices (ACIP) preferentially recommends three flu vaccines for people 65 years and older, namely, any one of the following higher-dose or adjuvanted influenza vaccines: quadrivalent high-dose inactivated influenza vaccine (HD-IIV4), quadrivalent recombinant influenza vaccine (RIV4), or quadrivalent adjuvanted inactivated influenza vaccine (aIIV4). Any other age-appropriate influenza vaccine should be used, if none of these three vaccines is available at an opportunity for vaccine administration [122]. Although comparisons of these vaccines with one another are limited, a greater potential benefit of HD-IIV3, aIIV3, or RIV4 relative to standard-dose unadjuvanted IIVs has been shown, with the most data available for HD-IIV3, in this age group. During the 2021/22 influenza season, vaccination programs were in place in all EU/EEA countries, most of which recommended vaccination against influenza for those aged ≥65 years (16 countries) (Table 2). Fewer countries reduced the lower age limit for vaccination at the age of ≥50, ≥55, ≥59, or ≥60 years or have recommendations for all adults ≥18 years (three countries). All countries had recommendations for individuals with chronic medical conditions and most for residents of long-term care facilities. In terms of vaccine type, quadrivalent vaccines (IIV4) are available in most EU/EEA countries, but nine countries offer other, potentially more effective vaccines for the elderly (e.g., aIIV4, HD-IIV4, and other augmented vaccines) (Table 2) [123].

### 3.3. RSV Vaccines and the Elderly

#### 3.3.1. Epidemiology and Clinical Manifestations of RSV Infection

Respiratory syncytial virus (RSV) is an enveloped negative-sense RNA virus that belongs to the *Paramyxoviridae* family. Minimal antigenic heterogeneity is displayed by RSV that is divided into two major subgroups (A and B), which differ antigenically in the P, N, F, and G proteins [124]. It is the most common cause of lower respiratory tract infections in infants and children and has been implicated in the development of reactive airway disorders such as asthma [125]. Most individuals are infected with the virus during the first years of life [126]. Since the resulting immunity is neither sustained nor complete, reinfections are common; however, they generally do not occur within the same season, suggesting a short-term immunity following natural infection [127].

RSV infections are associated with substantial morbidity in adults, with some analyses showing increased mortality from RSV compared to seasonal influenza [128,129]. Still, most infections in adults are mild and self-limiting, consisting primarily of upper respiratory tract symptoms, including cough, nasal congestion, sore throat, and a low-grade fever [130]. Cardiac, pulmonary, or renal comorbidities, in addition to immunocompromised states, are risk factors for severe RSV disease and lower respiratory tract involvement [131]. Adults over 65 years of age, regardless of comorbidities, are at a higher risk for severe RSV disease [132]. 

The impact of RSV in the elderly can be similar to that of seasonal influenza, with the most severe consequences in nursing homes, where annual attack rates reach 5–10%, with significant rates of pneumonia (10–20%) and death (2–5%) [133]. The in-hospital fatality rates related to RSV infections can be as high as 9.1% in developing countries [134]. Published estimates of RSV incidence and prevalence in older adults vary widely and are most likely underestimated [135]. RSV infections in the elderly are a significant cause of hospitalization. RSV is estimated to be responsible for approximately 214,000 (95% CI 100,000–459,000) hospitalizations annually, due to acute lower respiratory tract infections in adults ≥ 65 years of age in industrialized countries [136]. It is estimated that between 60,000 and 160,000 older adults in the US are hospitalized and 6000–10,000 die due to RSV infection each year [137]. Yet, accurately determining RSV morbidity and mortality in older adults is challenging, thus resulting in a substantial under-estimation of the disease burden [134]. Given the significant burden of RSV-related disease in the elderly, there is an urgent need to develop an effective vaccination strategy, which is why WHO has prioritized the development of an RSV vaccine, emphasizing the importance of vaccination in the adult age [138,139].

#### 3.3.2. Immunology of RSV Infection and Vaccine Design

Following natural infection from RSV, both antibody and cellular immune responses are mobilized to control the infection. Nonetheless, these responses are rather short-lived [140]. Antibody production (both in serum and in mucosal surfaces) protect against infection, whereas cellular immune responses appear to enhance viral clearance [141]. High levels of mucosal neutralizing antibodies protect adults from upper respiratory tract disease [142]. However, since RSV replicates solely within the respiratory tract, serum antibodies are not expected to prevent disease manifestation. High levels of serum-neutralizing antibodies may be associated with a reduced risk for lower respiratory tract infections [143], and this observation has served as the basis for the introduction of RSV-neutralizing antibody and monoclonal antibody treatments, currently used as passive immunization. Nevertheless, correlation does not imply causation since studies have shown that nasal IgA antibodies might be a better correlate of protection than serum antibodies [144,145]. Theoretically, a protective vaccine would be more efficient if it mimicked the natural route of infection and induced a robust mucosal antibody response [146], which is why mucosally administered vaccine platforms are continuously being studied for RSV [147]. 

A comprehensive literature review by Redondo et al. suggested that RSV vaccines should be integrated into routine immunization schedules and that an age-based strategy, i.e., recommending RSV vaccination for all individuals of a certain age regardless of their individual risk, should be prioritized over targeting high-risk groups [138]. Specifically, adults aged 60 years and older may benefit from RSV vaccination, especially those with chronic diseases, immunosuppression, or institutionalized status. Considering the geographical and seasonal variations of RSV, the optimal timing of vaccination should be determined according to local surveillance data but as early as possible for those eligible for vaccination. RSV vaccination should be offered to healthcare workers because of the higher risk of contracting and transmitting RSV to their patients [138].

#### 3.3.3. RSV Vaccines and Recommendations for the Elderly

Despite the many unknowns surrounding the correlates of protection against RSV infection [148], two RSV vaccines were recently approved for use in adults over the age of 60 [149]. Both are recombinant subunit prefusion F-protein-based vaccines, administered intramuscularly as a single dose. A recently published phase 3 trial showed that a single dose of an AS01_E_-adjuvanted RSV prefusion F protein-based vaccine (RSVPreF3 OA) was immunogenic and efficacious in preventing RSV-related lower respiratory tract disease (RSV-LRTD) and RSV-related acute respiratory illness (RSV-ARI) in older adults with cardiorespiratory and endocrine or metabolic conditions associated with an increased risk of severe RSV disease [150].

CDC guidelines recommend that these vaccine formulations be considered for all adults aged over 60, particularly those with comorbidities [151]. In Europe, only Austria and Sweden recommend vaccination against RSV for people older than 60 and 75 years, respectively [152]. Sweden recommends targeted vaccination for specific groups for people in the age group 60–74 years, while Belgium also recommends targeted vaccination for individuals older than 60 years. In none of these three countries is the cost of vaccination against RSV covered by the respective national health systems [152]. Both vaccines have shown a protective effect against lower-respiratory tract RSV infections [153,154]. The duration of the protective effect is still unknown; however, a protective effect was evident throughout the follow-up period for one of the two vaccines [153]. More observational data are needed to accurately determine the duration of protection.

### 3.4. COVID-19 Vaccines and the Elderly

#### 3.4.1. Epidemiology and Clinical Manifestations of SARS-CoV-2 Infection

SARS-CoV-2, an RNA virus of the *Coronaviridae* family, is the causal agent of the COVID-19 pandemic. As of March of 2024, more than 800 million people throughout the globe have been infected, and approximately 8 million have died as a direct result of the infection [155]. Although, in most instances, SARS-CoV-2 infection results in self-limiting symptoms, patients with risk factors, including underlying heart or lung disease, arterial hypertension, diabetes, immunosuppression, and the elderly, particularly older males, can develop severe disease requiring hospitalization and invasive mechanical ventilation [156]. 

The biomedical and pharmaceutical industry responded rapidly to the pandemic with the development of vaccines, which decreased transmissibility, disease severity, and overall mortality [157,158]. The development of antivirals followed, and therapeutics, particularly orally administered agents, have helped to keep vulnerable patients out of hospital [159]. As of May 2023, the WHO declared an end to the emergency phase of the COVID-19 pandemic [160]. As SARS-CoV-2 reinfections occur frequently in the post-Omicron era, a parallel pandemic of long COVID is unfolding, with considerable risk of various cardiac, pulmonary, or neurological complications, especially in the elderly population [161].

Advanced age is a risk factor for mortality from COVID-19. The presence of comorbidities and advanced age was found to be an independent risk factor for COVID-19 severity and hospitalization, as well as a predictor of poor prognosis [162]. It was recently revealed that ARDS and disseminated intravascular coagulation (DIC) complications and hospital length-of-stay were independent predictors of in-hospital mortality in elderly unvaccinated patients with COVID-19 [163]. In 2020, mortality from COVID-19 increased significantly after age 65, with the mortality rate for those over 80 reaching nearly 60% [164]. A recent study showed that the outcomes of COVID-19 among the old and oldest patients have improved compared to in the early phase of the pandemic due to widespread vaccination and advances in the treatment of COVID-19. Namely, in-hospital mortality was 14.6% among COVID-19 patients aged 65 or older in this study, showing no significant difference between the old and oldest subgroups, while comorbidities were still significantly related to poor hospital outcomes [162]. The risk of developing long COVID also increases with age, particularly in females [161,165,166]. Older people are at greater risk of persistent symptoms associated with COVID-19 compared to younger people. In addition, COVID-19 may cause or worsen chronic conditions that commonly occur in older people, such as cardiovascular diseases, diabetes, and other comorbidities [166]. 

#### 3.4.2. Immunology of SARS-CoV-2 Infection

Following infection with SARS-CoV-2, antibodies against viral proteins, particularly the S-protein, are induced, and overall antibody titers appear to be correlated with disease severity [167]. Although the virus encodes for four structural proteins, the spike (S), envelope (E), membrane (M), and nucleocapsid (N) [168], the S-protein is particularly immunogenic, and antibodies against the S-protein and especially those against the receptor binding domain (RBD) portion of the S-protein exhibit neutralizing properties [169], although their neutralizing capabilities likely diminish with subsequent viral variants such as those of the Omicron family [170]. High levels of neutralizing antibodies are associated with protection from infection [171], and in turn, anti-RBDs have been independently linked with a rise in neutralizing serum antibodies [172]. Therefore, all correlates of protection following vaccination focus primarily on measurement of anti-S and anti-RBD antibodies as a surrogate for the presence of neutralizing antibodies, and a high titer can be interpreted as (transient) immunity to infection.

#### 3.4.3. The Effect of Age on COVID-19 Vaccine Responsiveness—Pivotal Clinical Trials

In most pivotal randomized control trials, older individuals, particularly those who were frail and suffered from numerous comorbidities, were systematically underrepresented [173]. For example, in the phase III clinical trial of the Pfizer mRNA vaccine that demonstrated an efficacy of over 95% in preventing symptomatic disease, 42% of individuals were over 55, even fewer individuals were over 70 and only 5 individuals were over 75. These 5 individuals were also incidentally randomized on the placebo cohort and, therefore, did not receive the vaccine [174]. The initial study of the other mRNA vaccine licensed by Moderna, with 24.8% of enrolled individuals being over 65, demonstrated an 86.4% efficacy in those over 65, compared to an efficacy of 95.6% in those younger than 65 [175]. Subsequent analysis of the data for those over 65 showed an efficacy of 82.4% for those aged between 65 and 75. No COVID-19 cases were identified in those who received the vaccine and were aged over 75; therefore, no comparative data could be extracted for this age group [176]. 

Regarding the vector-based vaccine licensed by AstraZeneca, 22.4% of enrolled individuals were over 65, and the vaccine efficacy was 83.5% in those over 65, compared to 72.8% in those between 18 and 64. However, the incidence of SARS-CoV-2 infections in the over-65 age group was 0.1% in the vaccine arm and 0.8% in the placebo arm, compared to 0.5% in the vaccine arm and 1.7% in the placebo arm of the 18 to 64 age group [177]. In the single-dose vector-based vaccine licensed by Jannsen, 33.5% of individuals were over 60. The study revealed a vaccine efficacy of 76% in preventing moderate to severe COVID-19 in those over 60, two weeks after vaccine administration. The efficacy rate dropped to 66% after 28 days [178]. Finally, in the clinical trial assessing the efficacy of the recombinant, adjuvanted S-protein vaccine licensed by Novavax, 11.8% of individuals enrolled (approximately 2000 individuals) who received the vaccine were over 65 [179]. The overall vaccine efficacy was 90.4%. Age-specific vaccine efficacy rates were not provided since the efficacy for individuals over 65 was pooled with other high-risk chronic health conditions [179]. 

A recent cross-protocol analysis analyzed data from the initial studies of the Moderna, AstraZeneca, Janssen, and Novavax vaccines stratified for age and concluded that there was no significant difference in vaccine efficacy in preventing symptomatic and severe disease between older and younger adults [180]. However, the underrepresentation of older individuals in these pivotal vaccine trials presents an oxymoron since pre-vaccination laboratory data had implicated cellular and immune system senescence both with a higher risk for severe disease and a possible reduced vaccine efficacy [181,182]. In other words, it was well understood that older individuals and those with underlying chronic conditions would be the first to receive vaccines against COVID-19. 

#### 3.4.4. Vaccination and Duration of Immunity—Real-World Data and the Role of Boosters

Similarly to natural infection, immunity following COVID-19 vaccination wanes over time [183]. Feikin et al. analyzed data from 18 different studies prior to the emergence of Omicron and concluded that, 6 months post-vaccination, there was an overall reduction in vaccine efficacy of approximately 21%; the reduction in individuals over 50 was 20.7% [184]. However, the reduction in efficacy for protection against symptomatic COVID-19 was 24.9% overall and 32% in those over 50. Moreover, vaccine efficacy against severe COVID-19 was reduced by 10% overall and by 9.5% in those over 50 [184]. Sentogo et al., analyzing data from 18 studies that examined all vaccine formulations, reported that overall vaccine efficacy declined more rapidly and earlier in those over 65 compared to younger individuals [185]. The overall vaccine efficacy against severe COVID-19 was 90% and declined to 74% in those younger than 65 years of age 6 months post-vaccination and to 62% in those over 65 [185]. Other meta-analyses also supported the 6-month decline in vaccine immunity, following which protection against infection is sub-optimal and the decline in efficacy in those over 65 is steeper [186]. 

Booster shots may prolong protective antibody titers and protect elders and immunocompromised individuals from infection. Mattiuzi et al. compared data from approximately 2.4 million individuals over 80 years of age who completed the initial COVID-19 vaccination and 1.5 million who received booster shots and concluded that boosting resulted in an 75% reduction in risk for infection, 82% reduced risk for hospitalization, and 81% reduced mortality risk [187]. Yang et al., however, found that when compared to unvaccinated individuals over 80 years of age, vaccination does not seem to reduce mortality [188]. Vaccine efficacy in elders was nonetheless highly associated with booster shots, as evident by the stepwise increase in vaccine efficacy following three or four vaccine doses [188]. Li et al. analyzed data from 9 and 21 studies to determine efficacy and immunogenicity, respectively, of COVID-19 vaccines in older individuals aged over 55. Efficacy and immunogenicity were noted even in individuals over 65 years of age, and the authors concluded that the primary determinant of protection was the choice of initial vaccine, with mRNA vaccines being more effective, and the number of booster shots [189]. 

Similarly, a recent meta-analysis by Xu et al., which analyzed data from 22 pieces of randomized control data with a total of 3,404,696 adults aged over 60 years of age, indicated that COVID-19 vaccinations were effective in preventing infection and in reducing overall mortality in older individuals but not in reducing hospitalization rates or intensive care unit (ICU) admissions [190]. Moreover, mRNA vaccines were more immunogenic and more effective in eliciting a broad antibody response, with higher anti-S, anti-RBD, and neutralizing antibodies titers compared to other formulations [190]. Efficacy, as measured by mean antibody titers, was higher amongst the elderly vaccinated with any COVID-19 vaccine compared to a placebo, with no significant difference in titers with younger adults. Booster shots further augmented the duration and magnitude of the response [191].

#### 3.4.5. Putting It All Together

Current real-world data, as reviewed by numerous meta-analyses of the ever-expansive clinical trials and observational and retrospective studies, indicate that the primary COVID-19 vaccine cycles, as studied in the initial pivotal trials, are insufficient in providing protection from COVID-19 in older individuals. Booster shots are mandatory in order to bolster and extend the immune response. Furthermore, mRNA vaccinations may be more effective in inducing immune responses in older individuals.

However, assessing the efficacy of vaccines against COVID-19 in elders poses a significant challenge owing to the fact that many studies did not include elders or did not compare the magnitude of the immune response of elders with younger vaccine recipients. Moreover, assessing the efficacy of one vaccine formulation over the other from retrospective studies can be misleading because many individuals received booster shots in different time intervals and from different manufacturers [192]. Finally, as already mentioned, vaccine efficacy may be affected by circulating strains, as evident by numerous variants of the Omicron family, which fueled clinical research on newer vaccines against Omicron and Omicron-related strains [193]. Therefore, although meta-analyses and systematic reviews have provided information regarding the efficacy of COVID-19 vaccination in older individuals, any conclusion must be drawn with caution.

#### 3.4.6. Safety and COVID-19 Vaccination

Following the introduction of COVID-19 vaccines, safety concerns were voiced owing to their rapid deployment as a result of their emergency use status awarded by legislative and healthcare authorities. In addition, mRNA-based vaccines, a novel vaccine delivery platform, was deployed on such a massive scale for the first time. Numerous diverse adverse events, ranging from local injection-associated reactions or anaphylaxis [194] to cardiac complications, such as myocarditis and pericarditis [195], neurological insults including strokes [196], and acute kidney injury or renal failure [197], were reported infrequently.

The effect of age on the development of any vaccine-associated adverse event is challenging, owing to the inclusion of subjective symptoms and signs, such as dizziness and numbness, and the difficulty in establishing a clear causal relationship. This is especially true in older individuals with chronic conditions that might be the root cause of any possible new symptom. Numerous observational studies have associated a wide array of side effects with age. For example, Rosenblum et al. found that death was more common in those over 60, with a median age of 76, with no overall differences in reactogenicity when stratified for age [198], a finding also supported by Xiong et al. [199]. Jeong et al. showed that fever was more common in younger vaccine recipients [200]. Allergic reactions are more frequent in young females [201] and myocarditis in young males [202], but pericarditis affected predominantly males <40 and both sexes >40 years [195].

Systematic reviews have reported conflicting results regarding the elderly. Zhang et al. found that the adverse event rate amongst older vaccine recipients might be lower in older vaccine recipients than younger adults [191]. Meanwhile, Li et al. suggested that individuals over 65 years old were more likely to experience adverse events following any kind of vaccination [189].

#### 3.4.7. COVID-19 Vaccination Recommendations for Older Adults

On 28 February 2024, the CDC director, acknowledging the increased risk of severe disease from COVID-19 in older adults, endorsed the ACIP’s recommendation for adults aged 65 years and older to receive an additional dose of any updated (2023–2024 formula) COVID-19 vaccine (i.e., Moderna, Novavax, or Pfizer-BioNTech) at least 4 months following the last recommended dose of updated (2023–2024 formula) COVID-19 vaccine [203]. In the EU/EEA, there are no universal recommendations for vaccination against COVID-19 in older adults. Instead, the development of national vaccination strategies is supported by the EU Vaccines Strategy regarding the administration of vaccines, new and adapted, tailored to population groups in view of the possible emergence of new variants [204]. These strategies are to be based on the guidance issued by the European Medicines Agency and the European Centre for Disease Prevention and Control. 

Approximately 19.4 million people aged ≥60 years and 5.5 million people aged ≥80 years received one COVID-19 vaccine dose between 1 September 2023 and January 2024 in the EU/EEA. Median COVID-19 vaccination coverage rates of 11.1% (range: 0.01–65.8%) and 16.3% (range: 0.01–88.2%) among people aged ≥60 years and ≥80 years, respectively, with high variation among countries, were reported by 24 countries [205].

### 3.5. Pneumococcal Vaccines and the Elderly

#### 3.5.1. Epidemiology and Clinical Manifestations of Pneumococcal Disease

*Streptococcus pneumoniae*, a gram-positive bacterium, is responsible for a wide array of respiratory tract infections, including otitis, sinusitis, and pneumonia, but it can also cause systemic disease with the invasion of distal anatomical sites through the bloodstream, including endocarditis and meningitis [206]. Located on the surface of the bacterium, the polysaccharide capsule is an important virulence factor that protects the pathogen from phagocytosis by preventing complement opsonization [207]. The polysaccharide capsule exhibits an extraordinary variety, resulting in over 100 serotypes of *S. pneumoniae* [208]. Host immune responses may be serotype-specific; however, cross-serotype protection has been identified [209]. 

*Streptococcus pneumoniae* is responsible for significant morbidity and mortality in the elderly population [210]. The risk of acquiring non-invasive pneumococcal pneumonia (PP) and invasive pneumococcal disease (IPD) markedly increases with age [211]. The reported incidence of IPD in Europe varies widely, ranging from 0.4 cases to 20 cases per 100,000 population, reflecting both real differences and differences in diagnostic practice and the type, strength, and sensitivity of surveillance [206]. In 2018, the age-specific incidence of IPD in the EU/EEA was highest in the age group ≥ 65 years (18.7 confirmed cases per 100,000 population) [212]. In a population-based study conducted in Sweden, the 30-day case-fatality rate increased with age and was highest in the oldest age groups, particularly in patients with septicemia ≥75 years old (21.4%) [213]. 

The reported incidence of community-acquired PP is estimated at 1 per 1000 adults per year [206]; thus, the economic burden of treating this disease in adults ≥65 years is much higher compared to IPD [211]. Incidence rates and likelihood of serious outcomes of pneumococcal disease worsen with increasing frailty, multiple chronic diseases, particularly immunocompromising conditions, and immunosenescence [210,214]. It is known that the prevalence of comorbidities and immunocompromise increases with age, which contribute to the higher risk of pneumococcal disease [215]. Elderly people acquire pneumococcal disease due to community transmission of *S. pneumoniae*, usually through contact with visitors, family members, and caregivers, especially in nursing homes and other long-term care facilities [214]. Outbreaks of serious IPD (pneumonia, septicemia, and meningitis) have been reported in long-term care facilities, hospitals, and other close settings [206]. 

#### 3.5.2. Available Vaccines for Pneumococcal Disease

Two main types of vaccines exist against streptococcal disease: pneumococcal polysaccharide vaccines (PPVs) and pneumococcal conjugate vaccines (PCVs). 

Current market formulations of PPV contain 25 μg of purified pneumococcal capsular polysaccharides for 23 strains (PPV-23), whereas the PCV vaccine formulations contain saccharides approved against 13 (PCV-13) or, as of 2022, against 15 or 20 strains (PCV-15 or PCV-20). The PCV formulations contain polysaccharides conjugated with the CRM197 carrier protein [216]. PPV-23 confers protection against isolates 1, 2, 3, 4, 5, 6B, 7F, 8, 9N, 9V, 10A, 11A, 12F, 14, 15B, 17F, 18C, 19A, 19F, 20, 22F, 23F, and 33F, whereas PCV-13 confers protection against serotypes 1, 3, 4, 5, 6A, 6B, 7F, 9V, 14, 18C, 19A, 19F, and 23F. Two additional serotypes, 22F and 33F, were added into PCV-15. PCV-20 contains an additional five serotypes, 8, 10A, 11A, 12F, and 15B [217]. There is still debate regarding the optimal use of these vaccine formulations. Questions remain regarding the optimal vaccine schedule, their efficacy in preventing non-bacteremic disease, and the effect that infant and children vaccination may have on circulating pneumococcal strains that may affect vaccine efficacy.

Guidelines for pneumococcal vaccination differ between countries and continents for a myriad of reasons including differences in prevalence of disease in addition to reimbursement. For adults aged over 65, the CDC recommends the administration of one dose of either PCV-15 or PCV-20 with an additional booster shot of PPV-23 one year later, only if PCV-15 was administered [218]. This is based on evidence that demonstrated a booster response when PCV immunization was followed by a PPV dose. However, PCV administration after a PPV dose resulted in diminished immune responses [219,220,221,222]. Vaccine schedules and combinations between available formulations vary greatly between European countries [223].

#### 3.5.3. PPV—Protecting against Invasive or Non-Bacteremic Disease?

Invasive pneumococcal disease has been defined by most studies as the development of bacteremia, sepsis, or meningitis from *S. pneumoniae*, whereas non-bacteremic illness has been defined as an infection primarily located in the respiratory tract, such as pneumonia and otitis media [206]. 

A quadrivalent PPV was first tested in the middle of the 1940s and provided the first evidence of protection against pneumoccocal pneumonia. Inclusion of a greater repertoire of capsules resulted in the 23-valent PPV currently used today. Although initially thought to be vaccines that could prevent all pneumoccocal disease, subsequent studies cast doubts on the overall efficacy of these vaccines, primarily in preventing non-bacteremic streptococcal pneumonia [224,225]. In fact, a 2017 observational study of adults over the age of 65 showed that PPV-23 was 15% effective in preventing pneumonia and 28% effective in preventing ICU admission or death [226]. Moreover, a 2014 study demonstrated a 48% reduction in non-bacteremic streptococcal pneumonia and an overall reduction of 25% in all-cause community-acquired pneumonia [227]. 

The current literature has been explored by numerous authors in an effort to ascertain the overall efficacy of PPVs and especially their role in preventing pneumococcal pneumonia. Niederman et al. analyzed data from randomized control trials and real-word clinical studies and concluded that the PPV-23 formulation is effective in preventing invasive disease, especially in those younger than 75 years of age. Concerning non-bacteremic pneumonia, the formulation appears to confer protection for a span of approximately 5 years, and therefore, booster shots are necessary [228]. Falkenhorst et al. analyzed data from 17 studies and concluded that the PPV formulation was effective in reducing both invasive disease and non-bacteremic pneumonia, noting, however, that the data analyzed from clinical trials demonstrated increased efficacy due to the lower follow-up period, supporting the notion of a waning immune response following vaccination [229]. A systematic review by Diao et al. further concluded that PPV-23 formulations were associated with statistically insignificant findings in relation to reducing all-cause pneumonia (RR = 0.87), mortality due to pneumonia (RR = 0.67), and overall mortality (RR:1.04) [230].

Initial phase I and II clinical studies have demonstrated that PCV vaccines induce a more diverse and robust immune response compared to PPV [231]. Moreover, vaccine efficacy could be affected by prior anti-diphtheria antibodies, with individuals with higher anti-diphtheria antibodies inducing a more robust response following PCV administration [232]. Although few clinical trials have directly compared PPV and PCV, systematic reviews and meta-analyses have compared the efficacy of these formulations.

Dunne et al. analyzed data from nine studies and concluded that vaccine-type coverage against pneumonia was 2–6% for PPV-23 and 41–61% for PCV-13. The efficacy against streptococcal pneumonia or invasive disease was −10% to 11% for PPV-23 and 40 to 89% for PCV-13 [233]. Another meta-analysis by Sikjær et al. concluded that the overall PPV-23 efficacy against invasive pneumococcal disease was 28% to 54% in those aged 65–79, whereas PCV-13 efficacy was 75% in those over 65. However, it must be noted that, in this review, only one PCV-13 trial was considered eligible for inclusion [234]. 

Numerous mechanisms have been proposed as an explanation for the reduced efficacy of pneumococcal vaccines, primarily of PPV-23, in preventing non-bacteremic pneumonia. Non-response to specific serotypes of the 23 serotypes included in the formulation has been demonstrated with certain authors proposing that the PPV-23 vaccine is viewed as 23 different vaccines administered at the same time [235]. Moreover, it appears that *S. pneumoniae* cells that remain on mucosal surfaces, such as the respiratory epithelium, are frequently uncapsulated, in striking contrast to bacterial cells that enter the bloodstream [236]. Therefore, vaccines that induce an immune response against the streptococcal capsule are at a functional disadvantage in infections not involving the bloodstream.

The lowered efficacy against non-bacteremic pneumonia should not be interpreted solely as a failure of the vaccine formulation. Determining the infecting pathogen in cases of community-acquired pneumonia is a difficult challenge owing to the invasive methods necessary to obtain sufficient and quality material (such as bronchoalveolar lavage via bronchoscopy), which are required in order to reduce the risk of culture contamination from normal mouth flora during sputum expectoration. In many clinical studies, all-cause pneumonia can be used as a surrogate for pneumococcal pneumonia, assuming that *S. pneumoniae* remains one of the leading causes of bacterial pneumonia. However, the true incidence of true non-bacteremic pneumococcal pneumonia might be underrepresented in trials and clinical studies, due to the absence of an agreed-upon definition and of specific diagnostic criteria [237], thus underestimating vaccine efficacy. The differences in diagnostic definitions and criteria in addition to different study outcomes further complicate the systematic comparison of PCV and PPV formulations [238].

Finally, a relatively well-studied effect of PCV is the reduction in nasopharynx streptococcal carriage in children, an effect not observed with PPV [239]. This effect appears to enhance protection of unvaccinated elderly and frail individuals by inducing herd immunity. However, although the overall prevalence of vaccine-included serotype disease appears to decrease in countries with a high PCV vaccination rate, increased rates of non-vaccine-included serotypes appear to become ever more prevalent, a phenomenon termed serotype replacement [240]. Although the ramifications of this phenomenon have yet to be realized, such a phenomenon can affect overall vaccine efficacy and even alter vaccination guidelines.

## 4. Discussion

With the increased number of elderly patients, medical morbidities and healthcare spending are expected to rise [241]. The elders are a demographic of particular medical interest due to the numerous comorbidities in addition to an age-associated decline in immune system function. Consequently, infectious diseases, which primarily affect those with comorbidities and immune system impairment, particularly pneumonia, remain among the most common causes of death amongst the elderly. Infectious diseases in the elderly are associated with a higher mortality rate [242]. 

The aging body, with its altered cellular and physiological processes, has a profound effect on the efficacy and safety of most vaccine formulations. It has been shown that the immune response after vaccination, in terms of antibody titer, efficiency, and affinity of produced antibodies, as well as their longevity, decreases with aging [9]. The efficiency of available vaccines commonly remains low in the elderly population because of the poor ability of their immune systems to respond to immune stimulation [8]. The presence of multimorbidity and therapy with immunosuppressive effects (e.g., chemotherapy) can significantly weaken the vaccine-induced immune response and, thus, the protection offered by vaccines [3]. Improvement of existing vaccines and vaccination strategies, as well as development of new enhanced vaccines for existing or emerging pathogens, can significantly improve the health and quality of life of older adults [243]. 

In this review, we have presented current knowledge surrounding the most common vaccines used in older individuals and identified key research areas that could aid in enhancing the efficacy of available vaccine formulations. Efforts should be made to gain a comprehensive understanding of the mechanisms that lead to the aging of the immune system; the goal should be improving the coverage of recommended vaccines in the elderly and vaccination in periods when persons are immunologically competent before the onset of immunosenescence. All these strategies can be useful for the more effective protection of the elderly against infectious diseases both now and in the future [244].

Human immune responses following vaccination are affected by numerous factors including age, genetic predisposition, climate, and underlying medical conditions [12]. In this respect, homogenous trials are needed when trying to study the effect of each variable on the end result. Therefore, in order to assess the efficacy and safety of any vaccine formulation on elders, studies specifically geared to this demographic are needed. Most clinical studies and randomized trials nonetheless categorize respondents into broad age groups (e.g., over 65), possibly reducing confidence in reported results. This phenomenon can, in part, explain the conflicting results found in many meta-analyses and systematic reviews.

Research has continued to refine vaccines and develop new ones based on molecular technologies, such as the use of recombinant proteins and the development of mRNA vaccines that have been successfully used in older adults [3]. New technologies aimed at developing more effective vaccines in the elderly, based on targeting innate immunity to enhance the host’s immune response, are crucial to avoid the consequences of immunosenescence. It was recently established that innate immune cells can exhibit non-antigen-specific memory-like properties, thus exhibiting increased responsiveness to subsequent challenges with heterologous stimuli [8]. This concept of ‘trained immunity’ is a promising approach to improve the effectiveness of immunization in the elderly population. 

The most important benefit of vaccinations in the elderly is reflected in reduced morbidity and mortality from infectious diseases that can be prevented by vaccines. In addition, given the growing antibiotic resistance, successful vaccination indirectly contributes to the suppression of antibiotic resistance, potentially decreasing the need for antibiotics in older age. Although available vaccines provide some protective immunity in the elderly, they are still unable to provide long-term protection. Therefore, vaccines that induce long-lasting immune responses by strengthening cellular and mucosal immunity are essential. Key areas of research relate to vaccine formulations or platforms that include new vaccine adjuvants that specifically stimulate the magnitude and durability of the immune response, increasing the dose of antigen, ensuring that the risk of adverse events is not elevated, and exploring new routes of vaccine administration.

Vaccination recommendations must take into account the specific characteristics and burden of disease at the appropriate age, the properties of the available vaccine, and its immunogenicity and efficacy in older age groups, as well as underlying health conditions and the immune status of recipients. The aging of the immune system affects an individual’s immune response and often limits vaccine effectiveness. Therefore, further and carefully designed studies are needed to address the effects of immunosenescence and inflamm-aging to improve the elderly’s immune response to vaccination. 

High heterogeneity was noted amongst most meta-analyses, particularly for COVID-19 vaccination. Most reviews compared different studies with different vaccine formulations and with possibly different grading of disease severity (mild, moderate, and severe COVID-19) and of side-effects. The extreme variability in the timing and types of booster shots, as already mentioned, further perplexes the interpretation of results. Standardized definitions appear to be necessary in order to better clarify reports and data in this transitional post-emergency state of the COVID-19 pandemic. Hopefully, the lessons learnt during this pandemic will help us to be better prepared to protect especially the vulnerable older adults in a future pandemic.

This narrative review has several advantages. We conducted an extensive literature search to identify relevant studies and to highlight novelties in the most important vaccines for the elderly; furthermore, we consulted official websites to examine differences in vaccination recommendations for the elderly between the EU/EEA and the US. Results are described for each specific vaccine, filling a research gap, and opening the way for further research. Despite clearly defining the aim and criteria that allowed the studies included in the review to have comparable methodological quality, the heterogeneity of the studies limited us in reaching firm conclusions. This is probably the main limitation of this article. Another limitation of this work is that it focused on vaccines recommended for the elderly in Europe and the US. It would be interesting to investigate the global relevance of this article. Moreover, our bibliographic search was limited to the PubMed database and to articles written in English, and, therefore, other relevant articles only indexed in other databases may have been missed. Although the potential for publication bias is increased, we hope that this is an important step that helps in the design of more efficient and safe vaccines for seniors.

## 5. Conclusions

The conclusions of this article may be summarized into the following seven take-home messages:

1. Infectious diseases are among the most common causes of death in the elderly, especially in those with comorbidities and weakened immune systems.
2. Vaccination of the elderly reduces the risk of severe infections and of related hospitalizations and complications, as well as mortality rates associated with vaccine-preventable diseases.
3. By reducing the incidence of infectious diseases, vaccination of the elderly potentially reduces the need for antibiotics use, indirectly affecting bacterial resistance, while reducing treatment and other related economic costs.
4. Currently available vaccines are still unable to provide long-term protection. Despite reduced immunogenicity, vaccination of older adults may still provide significant benefits in terms of reducing the risk of severe illness, hospitalizations, and complications.
5. Vaccines that induce long-lasting immune responses by strengthening cellular and mucosal immunity are essential for the elderly.
6. There are significant differences between immunization policies, especially between European countries, but also between Europe and the US, in terms of recipient age, number of doses, and vaccination schedule and implementation (mandatory or recommended).
7. A consensus-based strategy in Europe could help to fill the gaps in immunization policy in the elderly, particularly regarding RSV and pneumococcal vaccination.

## Figures and Tables

**Table 1:** Recommended vaccination for herpes zoster in European Union and European Economic Area (EU/EEA) countries and the United States.

	Age (Years)							
	18	50	60	64	65	74	75	≥76
**EU/EEA** ^1^								
Austria								
Belgium								
Cyprus								
Czechia								
Estonia								
France								
Germany ^2^								
Greece ^3^								
Italy ^4^								
Liechtenstein								
Luxembourg ^5^								
Spain ^6^								
**United States** ^7^								

Light grey color: general recommendation; dark grey color: recommendation for specific groups only (e.g., the immunosuppressed); black color: recommended vaccination not funded by the national health system. ^1^ Bulgaria, Croatia, Denmark, Finland, Hungary, Iceland, Ireland, Latvia, Lithuania, Malta, the Netherlands, Norway, Poland, Portugal, Romania, Slovakia, Slovenia, and Sweden do not recommended vaccination for herpes zoster in older adults. ^2^ Germany recommends vaccination with 2 doses of the inactivated herpes zoster subunit vaccine, with a minimal and maximum interval of 2 to 6 months, respectively, between doses. ^3^ Greece recommends 2 doses (RZV vaccine) in immunocompromised with 2 or more episodes of herpes zoster, ZVL for those aged 60–75 years and RZV for the immunocompromised. ^4^ Italy recommends vaccination with 1 or 2 doses, depending on the vaccine used. ^5^ Luxembourg recommends vaccination with 2 doses 6–8 months apart. ^6^ Spain recommends vaccination with 2 doses of the inactivated herpes zoster subunit vaccine, with a minimal interval of 2 months between doses. ^7^ CDC recommends 2 doses of Shingrix (recombinant zoster vaccine, RZV) separated by 2 to 6 months, without having to screen, either verbally or by laboratory serology, for evidence of prior varicella [49,50].

**Table 2:** Recommended vaccinations for seasonal influenza in European Union and European Economic Area (EU/EEA) countries and the United States during the 2021–2022 influenza season (summarized from [123] for Europe and from [81] for the US).

	Age (Years)						Recommended Vaccines *
	≥18	≥50	≥55	≥59	≥60	≥65	
**EU/EEA**							
Austria							IIV4, aIIV4, QIV-HD
Belgium							IIV3, IIV4
Bulgaria							IIV4
Croatia							IIV4
Cyprus							aIIV4
Czechia							IIV4
Denmark							IIV4, QIV-HD
Estonia							IIV4
Finland							IIV4
France							IIV4, QIV-HD
Germany							IIV4, QIV-HD
Greece							IIV4
Hungary							IIV3
Iceland							IIV4
Ireland							IIV4, aIIV4
Italy							IIV4, aIIV4, cIIV4, rIIV4, QIV-HD
Latvia							IIV4
Liechtenstein							IIV4
Lithuania							IIV4
Luxembourg							IIV4
Malta							IIV4
The Netherlands							IIV4
Norway							IIV4
Poland							IIV4
Portugal							IIV4
Romania							IIV4
Slovakia							IIV4
Slovenia							IIV4
Spain							IIV4, aIIV3, aIIV4, cIIV4, QIV-HD
Sweden							IIV4, QIV-HD
**United States**							HD-IIV4, RIV4, aIIV4

Light grey color: recommended. * The funding of the vaccine and of the administration of the vaccine are covered in all EU/EEA countries except Liechtenstein. In Belgium, funding may be partial or total, depending on the age and risk group.

## Abbreviations

ACIP	(CDC’s) Advisory Committee on Immunization Practice
ARDS	Acute respiratory distress syndrome
BCG	Bacillus Calmette–Guérin vaccine
CDC	Centers for Disease Control and Prevention
DIC	Disseminated intravascular coagulation
ECDC	European Centre for Disease Prevention and Control
EEA	European Economic Area
EU	European Union
HA	Hemagglutinin
HZ	Herpes zoster (shingles)
ICU	Intensive care unit
IPD	Invasive pneumococcal disease
MMR	Measles, mumps, rubella
MMRV	MMR with varicella
NA	Neuraminidase
PP	Pneumococcal pneumonia
PCVs	Pneumococcal conjugate vaccines
PPVs	Pneumococcal polysaccharide vaccines
PHN	Post-herpetic neuralgia
RBD	Receptor-binding domain
RSV	Respiratory syncytial virus
RSV-ARI	RSV-related acute respiratory illness
RSV-LRTD	RSV-related lower respiratory tract disease
RZV	Recombinant zoster vaccine
ZLV	Zoster live vaccine
VZV	Varicella zoster virus
WHO	World Health Organization
